# Validation of the Copenhagen Psychosocial Questionnaire-Long Version II (COPSOQ II) in Greek employees

**DOI:** 10.14806/ej.26.1.977

**Published:** 2021-10-22

**Authors:** Eleni Zigkiri, Maria Charalampopoulou, Anastasia Kokka, Flora Bacopoulou, Christina Darviri, George P. Chrousos

**Affiliations:** 1 Postgraduate Course of Science of Stress and Health Promotion, School of Medicine, National and Kapodistrian University of Athens, Athens, Greece; 2 University Research Institute of Maternal and Child Health & Precision Medicine and UNESCO Chair on Adolescent Health Care, National and Kapodistrian University of Athens, Aghia Sophia Children’s Hospital, Athens, Greece

## Abstract

The aim of this study was to validate the long second version of the Copenhagen Psychosocial Questionnaire (COPSOQ II) in the Greek language. The study was carried out in two phases following a mixed-method design. Six hundred and fifty-two Greek employees (response rate 93.3%) responded in the second phase of the study, either online or in writing. Both types of participation to the study were confidential for the participants and the companies as well. Most participants were females (68.1%), married (47.4%), aged 30-49 years (60.9%), with high educational level (60.4%). The majority were occupied in the health and the social care services (37.4%) and employed by the private sector (63.2%). Internal consistency of the GR-COPSOQ II was assessed with the Cronbach alpha criterion (Cronbach’s a) and it was acceptable (0.8>α>0.7) for most of the scales. Mean scores were high in many scales that describe exposure to psychosocial risk factors at the workplace. Construct validity was established by exploratory factor analysis (EFA) for the entire study sample and scales. In conclusion, the Greek version of the COPSOQ-II (GR-COPSOQ II) has good psychometric properties and can be recommended as a valid tool for the assessment of psychosocial risk in Greek employees.

## Introduction

All over the world dramatic changes have been observed both in the workplace design and in the “social capital” of the enterprises (Berthelsen et al., 2016; Clausen et al., 2019; Jensen et al., 2017). The “fourth industrial revolution”, mostly related to the technological revolution, automatisation and computerisation, inevitably affects workplace organisation and may also affect the health and safety of workers (Leso et al., 2018). Greece, among other countries, has been under social economic reconstruction for more than ten years (Eikemo et al., 2018; Zartaloudis and Kornelakis, 2017) and many changes have been noticed in plenty of parameters regarding occupational status (Karadinos, 2013; Kentikelenis et al., 2011). Work design research has been based on contemporary and integrative clusters, and also on sporadically convenient work perspectives rather than substantive distinction. Work design, apart from the costs and benefits it entails, may also lead to a diverse set of outcomes (Parker et al., 2017)

Thus, it is important to find valid methods to assess workplace hazards and to ensure the health and safety of the workforce worldwide. One way to achieve that goal is by making the risk assessment easier and more cost-effective. Moreover, not only is it important to ascertain the risks, but it is equally important to predict as many as possible. Self-report questionnaires are a valuable tool for measuring multiple aspects of work characteristics (Beaton et al., 2000; Bland and Altman, 2002; Galanis, 2019; Guillemin et al., 1993; Laake et al., 2007; Maneesriwongul and Dixon, 2004). The assumption, which enables comparisons of results across different nationalities or countries, is the most valid perspective of a well standardised questionnaire among different languages (Erkut S, 2018; Prince, 2008; Weeks et al., 2007). The psychosocial work environment is crucial, among other work-related parameters, for the health and wellbeing of the workers and is defined as all those aspects of the work environment that pertain to interpersonal and social interactions, and influence behaviour as well as workplace development (Cox and Cox, 1993; Hupke, 2018; Jacobs et al., 2013; Niedhammer et al., 1998; Nuebling et al., 2013). Psychosocial workload can be measured either as an occupational risk factor or as a work related outcome. Moreover, it can be estimated indirectly by measuring vitality and mental health at the workplace (Burr et al., 2010), job satisfaction, general health, burnout, satisfaction with life (Nuebling et al., 2013), absenteeism (Clausen et al., 2015, 2019; Clausen and Borg, 2010), and commitment at the work place (Clausen and Borg 2010; Clausen et al., 2015).

Among other instruments, the Copenhagen Psychosocial Questionnaire (COPSOQ) is one of the most widely used self-report questionnaires for the assessment of the occupational psychosocial risk factors. COPSOQ has been developed for the needs of the studies of the Danish National Research Centre for the Working Environment, from 1995, in Denmark (Kristensen et al., 2005). The COPSOQ authors, Tage Kristensen and Vilhelm Borg, kept on the development of the first version by getting feedback (*e.g.* lack of access to domains such as justice, reward, trust and discrimination). In 2010, the second version of the questionnaire (COPSOQ II) was published (Bjorner and Pejtersen, 2010; Pejtersen et al., 2010). Both questionnaires are available in three versions (long, medium and short) and their usage depends on the purpose (long for surveys, middle and short in accordance with the workplace and the work environment professionals). The response options on a Likert type four or five answer scale range from 0 to 100 points. The higher scores represent high level of the concept being measured. The average scores of the included items are the overall score for the seven main domains of the instrument. Six items have to be reversed before scoring (QD4: Do you have enough time for your work task? Va 2: Do you have to do the same things over and over again? CW4: How often do you consider looking for work elsewhere? TE1: Do the employees withhold information from each other? TE2: Do the employees withhold information from the management? TM 3: Does the mangement withhold important information from the employees?). The duration for answering the questionnaire is about 20 minutes to half an hour and the total score of each scale is the average of the items scored. If less than half of the items of each scale are not answered by a respondent the subject is consided as missing for that scale.

The COPSOQ is recognized as an example of good practice by the European Occupational Safety and Health Agency (EU OSHA) and it is cited in many documents of international organisations. The COPSOQ II has already been translated in more than twenty-five languages (Alvarado et al., 2012; Berthelsen et al., 2016, 2017, 2018; Dicke et al., 2018; Dupret et al., 2012; Isha et al., 2020; Moncada Lluís et al., 2008; Moncada et al., 2014; Nübling et al., 2006; Pournik et al., 2015; Ramkissoon et al., 2019; Shan *et al.*, 2008. Moreover, it has been used for comparisons among countries. The COPSOQ appears in hundreds of references in indexed international scientific journals on Medline.

The third version of the COPSOQ has been structured (Burr et al., 2019) and the whole procedure was coordinated by the International COPSOQ Network^1^.

The aim of this study was to test the psychometric equivalence and validate the Greek translation of the long version of the Copenhagen Psychosocial Questionnaire-second version (COPSOQ II), in a sample of Greek employees.

## Materials, Methodologies and Techniques

The study was conducted from September 2018 to December 2019 in Greece, following a thourough search of the international bibliography for an instrument suitable to evaluate various aspects of the work environment and not only some parameters of work-related stress risk factors and their outcomes.

The study protocol was approved by the ethics committee of the National and Kapodistrian University of Athens and the research was carried out in two phases. In the first phase, the translation and the cross-cultural adaptation of the questionnaire was performed, and in the second phase the psychometric properties of the questionnaire were evaluated in a representative number of Greek employees.

Regarding some researchers, a number between five to ten participants is considered satisfactory for the confirmation of the reliability and the validity of a questionnaire (McDermott and Palchanes, 1994; Harkness and Zentrum, 1998). Thus, a minimum number of 640 (128*5) cases/respondents or valid questionnaires would probably fit well for the purpose of this methodological study.

### Process of COPSOQ II translation in the Greek language

Following the typical procedure, permission to validate the questionnaire was obtained by its creators Professors Vilhelm Borg and Tage Kristensen, in December 2017.

**Figure 1.**
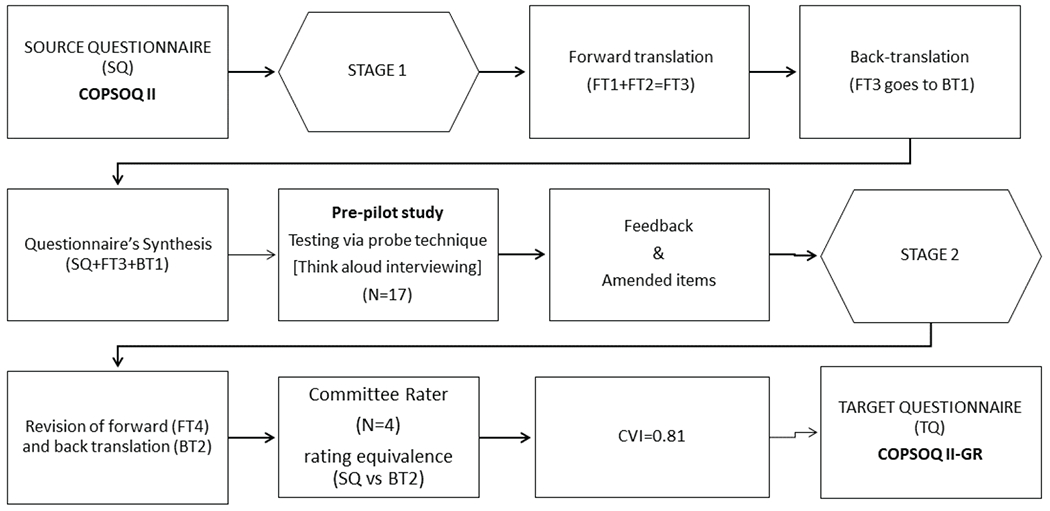
Linquistic adaptation process of the COPSOQ II, in the Greek language.

A mixed-method design, combining a qualitative study with probe technique characteristics (*e.g.* interviews, a committee rater judgement and CVI score) and a quantitative procedure was utilized. The instrument (COPSOQ II) for the evaluation of the psychosocial work-related risk factors among Greek employees was developed in two phases:

In the first phase, the overall item development, forward / back-translation, and cross-cultural adaptation of the questionnaire was tested by the content validity criterion (Figure 1), and in the second phase, the equivalence of other psychometric properties, such as construct validity, internal consistency and reliability were tested.

The pilot study of the questionnaire was performed in 120 workers (response rate 85%) and showed good psychometric properties, with Cronbach-alphas more than 0.70 on most scales (“Demand at Work” α=0.87, “Work Organization and Job Contents” α= 0.90, “Interpersonal Relations and Leadership” α= 0.85, “Values at Workplace” α= 0.86, Health and Well-Being” α= 0.92, “Offensive Behaviour α=0.85). The only scale with a slightly lower Cronbach alpha was the “Work Individual Interface” scale (α= 0.68).

Questions about demographic characteristics such as age, sex, marital status, occupational sector, number of employees and employment status were also included.

### Greek version of the COPSOQ II

The final version of the Greek questionnaire was shared in two different ways (online and in writing). Regarding paper documentation, the questionnaire, the consent form, the cover letter explaining the purpose of the study, as well as researchers’ affiliation, were enclosed in the same envelope. The file was handed over to employees who belonged to as many different occupational sectors as possible. Whenever a company or a stricter occupational sector had any doubts about the participation of their employees in the survey, the procedure was necessarily more thorough. In this case, a written approval of the scientific committee or the HR department of the company was also requested.

Regarding the online form of the questionnaire, the Research Electronic Data Capture (REDCap) application had been selected to build the electronic form of the Greek version of the COPSOQ II. REDCαp is a secure web application for building and managing online surveys and databases. While REDCap can be used to collect virtually any type of data in any environment (including compliance with 21 CFR Part 11, FISMA, HIPAA, and GDPR), it is specifically geared to support online and offline data capture for research studies and operations. REDCap is a web-based application developed by Vanderbilt University to capture data for clinical research and create databases and projects. It is Health Insurance Portability and Accountability Act (HIPAA)–compliant, highly secure, and intuitive to use (Harris et al., 2009, 2019; Patridge and Bardyn, 2018).

Both types of participation to the study were confidential for the participants and the companies as well.

### Psychometric properties

#### Reliability analysis:

The internal consistency analysis of the GR-COPSOQ II was assessed with the Cronbach alpha criterion (Cronbach’s a). The original Danish study (Pejtersen et al., 2010) and other validation studies of the COPSOQs (Rosário et al., 2017) adjust the conversional threshold of 0.70 as an acceptable value for Cronbach. Moreover, a≥0.90 is an excellent value and a≤0.50 is an unacceptable value for the internal consistency of the scale, which is accessing.

#### Construct validity:

Construct validity was established by expolratory factor analysis (EFA) for the entire study sample (N = 652). Expolratory Factor Analysis (EFA) was used to cross-validate the derived factor structures. Values for Kaiser–Meyer–Olkin (KMO) measure of sampling adequacy and Bartlett’s test of sphericity (preferably significant) were used to assess the suitability of data for factorisation. The criterion for loading and cross loading was set at 0.4. Items loading below 0.40 and cross loading over 0.40 might be necessary to be deleted.

### Statistical Analysis

Statistical analysis was carried out by using SPSS version 25 (IBM Corp. Released 2017. IBM SPSS Statistics for Windows, Version 25.0. Armonk, NY: IBM Corp). For the quantitative variables of the questionnaire (GR-COPSOQ II) data were presented as a mean and standard deviation (mean ± SD), while for the qualitative variables data were presented as frequencies (n) and percentages (%). The Cronbach -alpha criterion was used for internal consistency and values higher than 0.7 were considered as appropriate (DeVellis, 2016). The missing values were treated by listwise pairs deletion (Soley-Bori, 2019). Construct validity and more specifically factor analysis was examined by undertaking Principal-Component Exploratory Factor Analysis with a varimax rotation.

## Results

The sample of the basic study consisted of 652 Greek employees (93.3% response rate) who completed the Greek version of the GR-COPSOQ II questionnaire. Descriptive statistics of the demographic and other characteristics of the sample are presented in Table 1 in Supplementary Data^2^. One third of the participants were male and 63.2% of the participants were from the private sector. The majority of the sample was married (47.4%), aged 30-39 years (31.1%), with high educational level (60.4%). Most of them declared that they were occupied at the health and the social care sectors (37.4%), in big companies with over than 500 employees (28.5%) or small with fewer than 20 employees (25.7%), and they work more than 40 working hours per week (33.8%). Also, they had 10-20 years of working experience (37.9%).

The internal consistency analysis of the GR-COPSOQ II was calculated with the Cronbach alpha criterion (Tables 2 and 3 in Supplementary Data^2^). The original Danish study (Pejtersen et al., 2010) and other validation studies, *e.g.*, Portuguese (Rosário et al., 2017), adjust the conversional threshold of 0.70 as an acceptable value for Cronbach (DeVellis, 2016).

For the pilot study, all Cronbach’s alpha indicators for domains ranged from 0.68 to 0.92 and four scales ranged from 0.58 to 0.95 (the lower value of alpha is due to the fact that this scale consists of only two items). Twenty-six out of 33 scales with Cronbach’s alpha, were found to have higher alpha in the Greek version than the original version, six of them had lower alpha and one scale had equal alpha. Compared to the Portuguese version, only three scales had lower alpha in the Greek version and one had equal value.

In the main study, domains’ Cronbach’s alpha indicators ranged from 0.57 to 0.91. More specifically, for “Demand at Work” Cronbach’s alpha was computed as a=0.86 (scales ranged from α=0.66 to α=0.71), for “Work Organization and Job Contents” Cronbach’s alpha was computed as α=0.89 (scales ranged from α=0.31 to α=0.81), for “Interpersonal Relations and Leadership” Cronbach’s alpha was computed as α=0.86 (scales ranged from a=0.49 to α=0.92), for “Work Individual Interface” Cronbach’s alpha was computed as α=0.57 (scales ranged from α=0.68 to α=0.87), for “Values at Workplace” Cronbach’s alpha was computed as α=0.83 (scales ranged from α=0.64 to α=0.86), for “Health and Well-Being” Cronbach’s alpha was computed as α=0.91 (scales ranged from α=0.76 to α=0.88), and for “Offensive Behaviour” Cronbach’s alpha was computed as α=0.86.

The mean scores and standard deviations are shown in Table 3 in Supplementary Data^2^, where they are compared with the original Danish study (Pejtersen et al., 2010) and the Portuguese study (Rosário et al., 2017)

Moreover in Table 3 in Supplementary Data^2^, scales in the Greek study with negative scoring, where a high score means “bad” or “unhealthy” (*i.e.* Quantitative Demands, Cognitive Demands, Emotional Demands, Work Pace, Demands for Hiding Emotions, Role Clarity, Role Conflicts, Work-Family Conflict, Family-Work Conflict, Burnout, Stress, Sleeping Problems, Depressive Symptoms, Somatic Stress and Cognitive Stress), showed increased average values compared to the Danish and the Portuguese study. There was an exception in the scale of “Job Insecurity”, whereas in the Danish study there was a lower average value in contrast to the Portuguese and the Greek study. Greek study’s scales included in “Health and Well-Being” domain showed higher means scores in comparison with the original and the Portuguese study, except for those with a positive meaning such as Self-efficacy.

However, ten scales (*i.e.*, Variation of Work, Meaning of Work, Commitment to the Workplace, Predictability, Recognition-Rewards, Quality of Leadership, Social Support from Supervisors, Job Satisfaction, Mutual Trust between Employees, Trust Regarding Management, Justice and Social Inclusiveness) for which high score means “good” or “healthy”, the Greek study showed lower average values. Otherwise, in “Influence at Work” and in “Social Support from Colleagues”, the Greek study showed higher average values. In scales “Possibilities for Development” and “Social Community at Work”, the values of the Greek studystand between the corresponding values of the Danish and Portuguese study.

Furthermore, Greek employees showed increased proportions in the domain of “Offensive Behaviour” that included meanings such as “Sexual Harassment”, “Threats of Violence”, “Bullying, Unpleasant Teasing”, “Conflicts and Quarrels”, “Gossip and Slander”. Only in “Physical Violence”, the Danish study showed a small difference compared to the Greek study.

An exploratory factor analysis was conducted considering the seven dimensions of the long version of the COPSOQ II, and the results are summarized in Tables 4, 5, 6, 7, 8, 9, and 10 in Supplementary Data^2^.

In the Demands domain at “Work Dimension” the results support the scales (Quantitative Demands, Work Pace, Cognitive Demands, Emotional Demands and Demands for Hiding Emotions). In the scale of “Cognitive Demands”, two items loaded in two factors, but still this factor can be supported sufficiently (Table 4 in Supplementary Data^2^).

In the “Work Organisation and Job Contents” dimension (Table 5 in Supplementary Data^2^), the results support totally the “Influence at Work”, “Possibilities for Development”, “Meaning of Work” scales. “Variation of Work” scale and “Commitment to the Workplace” scale are split into two factors, indicating that the construct validity of this scale is not supported. The last item of “Commitment to the Workplace” scale showed a negative loading indicating that this item should be reversed ,as should be done in the initial study.

In the “Interpersonal Relations and Leadership” dimension (Table 6 in Supplementary Data^2^), factor analysis showed six factors instead of the initial eight factors. The analysis support the “Recognition-Rewards”, “Role clarity”, “Quality of Leadership”, “Social Support from Colleagues”, “Social Support from from Supervisors” and “Social Community at Work” scales. Four scales (“Social Support from Colleagues” and “Social Community at Work”) and (“Quality of Leadership” and “Social Support from Supervisors”) load on the same factor. Two other scales (“Predictability” and “Role Conflicts”) load on various scales.

In the “Work-individual Interface” dimension, the results fully support the initial scale structure (Table 7 in Supplementary Data^2^) as in the Danish study.

In the “Values at the Workplace” dimension, the results did not support the hypothesised scale structure for any of scales. At least one item for each scale loaded in different factor while three or more items loaded in the same factor (Table 8 in Supplementary Data^2^).

In the “Health and Well-Being” dimension, the results support the hypothesised scale structure for three scales (“Burnout”, “Depressive Symptoms” and “Cognitive stress”) but in this factor analysis six factor were extracted instead of the initial eight scales. The “Sleeping Problems”, “Somatic Stress” and “Self-efficacy” scales had one item which loaded in a different factor. In the “Stress” scale, three of the four items loaded in two factors (Table 9 in Supplementary Data^2^).

In the “Offensive Behaviour” dimension (Table 10 in Supplementary Data^2^), factor analysis resulted into two factors; one with “Sexual Harassment”, “Bullying”, “Unpleasant Teasing” and “Gossip and Slander”, and one with “Threats of Violence”, “Physical Violence” and “Conflicts and Quarrels”.

## Discussion

For almost a decade, the Greek working force experiences chronic stressors due to the sustained socio-economic changes (Kentikelenis et al., 2011). To detect the impact of that stressors in the workplace and improve working conditions, proper tools are necessary to monitor work-related stress. Psychosocial risk factors are work-related chronic stressors with substantial impact on the social capital and the working environment itself (Leka and Jain, 2010).

The Copenhagen Psychosocial Questionnaire is one of the most valid tools used for assessment of the psychosocial workload almost worldwide (Dicke et al., 2018; Nübling *et al.*, 2014).

This study provides evidence that the Greek version of COPSOQ-II is valid and reliable. The long version of the questionnaire was tested through different techniques (probe techniques with an evaluation by four committee raters, item CVI score, S-CVI/average and content validity), at the first phase of the study, with the purpose not only to be translated into Greek but also to be cross-culturally adapted. The final adaptation (study’s second phase) was conducted on a sample of 652 workers from various occupational sectors. The Greek version of COPSOQ-II revealed satisfactory psychometric properties.

The internal consistency of the seven domains was satisfactory (> 0.80) with the exception of the “Work individual interface” domain that showed low internal consistency. However, the specific subscales of this domain showed high internal consistency. This may be a matter of further investigation for testing with other criterion equivalence, *e.g.*, convergent validity.

Comparison of the Greek (GR) version Cronbach-alphas with the results of the subscales in the original survey (Danish - DN) and in another country with similar socioeconomic conditions (Portuguese - PT), showed similarities. However, internal consistency was observed in the following subscales for GR and PT vs. DN; “Predictability”: GR (α=0.50), PT(α=0.49) vs. DN (α=0.74), “Mutual Trust between Employees”: GR (α=0.69), PT (α=0.66) vs. DN(α=0.77), “Job Satisfaction”: GR (α=0.72), PT (α=0.72) vs. DN (α=0.82). These might be explained by the fact that Denmark’s labour market is established on the basis of “flexicurity” (Richard *et al.*, 2012) that leads to more predictable working conditions, while Greece and Portugal are under the control of the International Monetary Fund (IFM) ( Karadinos, 2013; Kentikelenis et al., 2011).

Regarding mean scores of the several subscales (“Quantitative demands”, “Cognitive demands”, “Emotional Demands”, “Work Pace”, “Demands for Hiding Emotions”, “Role Clarity”, “Role Conflicts”, “Work-Family Conflict”, “Family-Work Conflict”, “Burnout”, “Stress”, “Sleeping Problems”, “Depressive Symptoms”, “Somatic Stress “ and “Cognitive Stress”), Greek employees scored higher than the Portuguese and the Danish. This may be attributed to the fact that our study was conducted at a period of time where the Greek working force had been exposed to increased psychosocial workload for almost 10 years, depicting the exhaustion from the ongoing changes that had taken place in their work.

The results from the factor analysis are partially consistent with the initial version of the COPSOQ-II, as three of the subscales (“Interpersonal Relations and Leadership”, “Health and Well-Being”, “Offensive Behaviour”) resulted in less factors. The results of the factor analysis regarding the domains of “Interpersonal Relations and Leadership” and “Offensive Behaviour” may be explained by the fact that many participants were occupied in positions free of leaderships, supervisors, or colleagues. Health and well-being domain also resulted in less factors and possible explanations are the sample size, the inability of some participants to comprehend the items of the subscales, or the difficulty to interrelate items within this scale.

One of the strengths of the study is that it included employees from different sectors. Also, the sample size was adequate and the internal consistency of the subscales was satisfactory. However, due to the long version of the questionnaire, no test-retest reliability was performed, which is a limitation of the study.

In conclusion, the Greek version of the COPSOQ-II (GR-COPSOQ II) indicated good psychometric properties for most of the scales of the questionnaire. The GR-COPSOQ II can be recommended as a valid tool for the assessment of psychosocial risk factors in Greek employees as it meets the criteria of internal consistency and construct validity.

## Supplementary Material

Tables 1-10 in Supplementary Data

## Key Points

- Work-related stress can manifest with a series of psychosocial, emotional, cognitive and behavioural reactions.
- Validated instruments are required to measure psychosocial risk factors in the work environment.
- The Copenhagen Psychosocial Questionnaire-II (COPSOQ-II) is a widely used tool.
- Validity and reliability of the COPSOQ-II were tested in a large sample of Greek employees.
- The Greek version of the COPSOQ-II has good psychometric properties and is recommended for psychosocial risk assessment in Greek employees.

## Footnotes

1
https://www.copsoq-network.org/

2
http://journal.embnet.org/index.php/embnetjournal/article/downloadSuppFile/977/977_supp_1
