# Supplementary material for: Validation of the Copenhagen Psychosocial Questionnaire-Long Version II (COPSOQ II) in Greek employees
Source: EMBnet J. Author manuscript; Available in PMC 2022 May 19. (PMC9119015; doi:10.14806/ej.26.1.977)
Supplement: Tables 1-10 in Supplementary Data [file NIHMS1766880-supplement-Tables_1-10_in_Supplementary_Data.docx]

**Table 1.** Participants’ demographic characteristics (Ν = 652).

| Category | Descriptive | *n*  (%) |
| --- | --- | --- |
| Sex | Male  Female | 208(31.9)  444 (68.1) |
| Age group (years) | <20  21-29  30-39  40-49  50-59  60-64  >65 | 19 (2.9)  108 (16.6)  203 (31.1)  194 (29.8)  107 (16.4)  15 (2.3)  6 (0.9) |
| Marital status | Single  Married  Divorced  Cohabitation  Widower | 213 (32.7)  309 (47.4)  67 (10.3)  46 (7.1)  17 (2.6) |
| Educational level | Basic  Low  Medium  High | 66 (10.1)  98 (15.1)  93 (14.3)  392(60.4) |
| Economic activities | Health and Social work  Education and Academic  Catering and Service  Commercial  Companies  Other | 241 (37.4)  77 (11.9)  83 (12.8)  40 (6.2)  108 (16.7)  96 (14.9) |
| Sector | Private  Public  Freelancers | 387 (63.2)  153 (25.0)  72 (11.8) |
| Number of employees | <20  20-50  50-100  100-500  >500 | 145 (25.7)  59 (10.5)  99 (17.6)  100 (17.7)  161 (28.5) |
| Number of colleagues | <5  10-20  20-40  >40 | 258 (44.9)  221 (38.4)  67 (11.7)  29 (5.0) |
| Work experience  (years) | <1  1-9  10-20  20-30  >30 | 11 (1.7)  126 (19.6)  244 (37.9)  185 (28.8)  77 (12.0) |
| Employment contract | Full-time  Hourly wages  Seasonal | 467 (73.5)  91 (14.3)  77 (12.1) |
| Working hours/week | <10  11-20  21-30  31-40  >40 | 62 (9.8)  52 (8.2)  113 (17.9)  192 (30.3)  214 (33.8) |
| Values refer to frequencies (n) and percentages (%).  Educational level was categorized as basic (high school graduates), low (technical school graduates), medium (Technological Educational Institution graduates), high (University graduates, Master and PhD holders). | | |

**Table 2.** Cronbach’s alpha values of the domains of the Greek version.

| Domains | CRONBACH α | Items |
| --- | --- | --- |
| Demand at Work | 0.86 | 18 |
| Work Organization and Job Contents | 0.89 | 17 |
| Interpersonal Relations and Leadership | 0.86 | 25 |
| Work Individual Interface | 0.57 | 14 |
| Values at Workplace | 0.83 | 15 |
| Health and Well-Being | 0.91 | 31 |
| Offensive Behaviour | 0.84 | 7 |

**Table 3.** Reliability and descriptive statistics’ comparisons between the original version (DK), the Portuguese version (PT) and the Greek version (GR).

|  |  | Cronbach’s α | | | Mean ± SD a | | |
| --- | --- | --- | --- | --- | --- | --- | --- |
| Domains | **Scales** | **DK** | **PT** | **GR** | **DK** | **PT** | **GR** |
| Demands at Work | Quantitative Demands [4] | 0.82 | 0.69 | 0.73 | 40.2 ± 20.5 | 36.3 ± 18.2 | 46.8 ± 20.9 |
|  | Work Pace [3] | 0.84 | 0.74 | 0.76 | 59.5 ± 19.1 | 63.1 ± 19.2 | 67.7 ± 20.5 |
|  | Cognitive Demands [4] | 0.74 | 0.63 | 0.72 | 63.9 ± 18.7 | 57.0 ± 18.4 | 69.5 ± 19.0 |
|  | Emotional Demands [4] | 0.87 | 0.73 | 0.81 | 40.7 ± 24.3 | 54.9 ± 20.8 | 59.9 ± 22.4 |
|  | Demands for Hiding Emotions [3] | 0.57 | 0.60 | 0.66 | 50.6 ± 20.8 | 39.7 ± 23.3 | 73.6 ± 19.8 |
| Work Organization and Job Contents | Influence at Work [4] | 0.73 | 0.53 | 0.72 | 49.8 ± 21.2 | 47.2 ± 19.0 | 52.5 ± 23.2 |
|  | Possibilities for Development [4] | 0.77 | 0.71 | 0.81 | 65.9 ± 17.6 | 68.7 ± 17.0 | 67.0 ± 22.7 |
|  | Variation of Work [2] | 0.50 | 0.23 | 0.31 | 60.4 ± 21.4 | 50.4 ± 19.8 | 50.2 ± 19.9 |
|  | Meaning of Work [3] | 0.74 | 0.70 | 0.77 | 73.8 ± 15.8 | 75.9 ± 17.7 | 72.7 ± 22.2 |
|  | Commitment to the Workplace [4] | 0.76 | 0.61 | 0.69 | 60.9 ± 20.4 | 69.5 ± 16.4 | 59.9 ± 21.7 |
| Interpersonal Relations and Leadership | Predictability [2] | 0.74 | 0.50 | 0.49 | 57.7 ± 20.9 | 58.6 ± 19.5 | 56.2 ± 23.5 |
|  | Recognition-Rewards [3] | 0.83 | 0.67 | 0.92 | 66.2 ± 19.9 | 66.9 ± 19.2 | 56.6 ± 29.4 |
|  | Role Clarity [3] | 0.78 | 0.72 | 0.82 | 73.5 ± 16.4 | 60.2 ± 14.7 | 78.1 ± 20.4 |
|  | Role Conflicts  [4] | 0.67 | 0.70 | 0.64 | 42.0 ± 16.6 | 44.2 ± 19.0 | 50.0 ± 21.7 |
|  | Quality of Leadership [4] | 0.89 | 0.90 | 0.84 | 55.3 ± 21.1 | 64.6 ± 21.5 | 49.4 ± 25.2 |
|  | Social Support from Colleagues [3] | 0.70 | 0.65 | 0.74 | 57.3 ± 19.7 | 59.6 ± 21.9 | 63.5 ± 20.2 |
|  | Social Support from Supervisors [3] | 0.79 | 0.84 | 0.81 | 61.6 ± 22.4 | 68.4 ± 19.2 | 60.3 ± 23.8 |
|  | Social Community at Work [3] | 0.85 | 0.81 | 0.83 | 78.7 ± 18.9 | 59.3 ± 20.3 | 69.7 ± 21.2 |
| Work-Individual Interface | Job Insecurity  [4] | 0.77 | 0.77 | 0.68 | 23.7 ± 20.8 | 43.9 ± 26.1 | 32.3 ± 22.9 |
|  | Job Satisfaction [4] | 0.82 | 0.72 | 0.73 | 65.3 ± 18.2 | 62.5 ± 16.0 | 62.4 ± 19.1 |
|  | Work-Family Conflict [4] | 0.80 | 0.84 | 0.81 | 33.5 ± 24.3 | 40.0 ± 26.7 | 57.8 ± 26.3 |
|  | Family-Work Conflict [2] | 0.79 | 0.76 | 0.87 | 7.6 ± 15.3 | 10.7 ±16.9 | 22.8 ± 28.9 |
| Values in the Workplace | Mutual Trust between Employees [3] | 0.77 | 0.66 | 0.69 | 68.6 ± 16.9 | 69.0 ± 16.6 | 54.9 ± 21.5 |
|  | Trust Regarding Management  [4] | 0.80 | 0.60 | 0.71 | 67.0 ± 17.7 | 62.8 ± 18.2 | 53.3 ± 20.1 |
|  | Justice [4] | 0.83 | 0.81 | 0.86 | 59.2 ± 17.7 | 61.8 ± 18.3 | 50.2 ± 24.3 |
|  | Social Inclusiveness [4] | 0.63 | 0.65 | 0.64 | 67.5 ± 16.3 | 59.0 ± 20.7 | 56.7 ± 22.8 |
| Health and Well-Being | General Health Perception [1] | – | – | – | 66.0 ± 20.9 | 58.3 ± 22.8 | 60.6 ± 26.0 |
|  | Burnout [4] | 0.83 | 0.91 | 0.87 | 34.1 ± 18.2 | 32.9 ± 22.5 | 56.5 ± 22.3 |
|  | Stress [4] | 0.81 | 0.83 | 0.88 | 26.7 ± 17.7 | 43.9 ± 22.3 | 55.2 ± 21.1 |
|  | Sleeping Problems [4] | 0.86 | 0.88 | 0.77 | 21.3 ± 19.0 | 38.7 ± 21.6 | 39.3 ± 21.7 |
|  | Depressive Symptoms [4] | 0.76 | 0.77 | 0.82 | 21.0 ± 16.5 | 32.9 ± 22.5 | 37.0 ± 22.5 |
|  | Somatic Stress [4] | 0.68 | 0.70 | 0.76 | 17.8 ± 16.0 | 26.9 ± 18.9 | 39.1 ± 21.6 |
|  | Cognitive Stress [4] | 0.83 | 0.84 | 0.86 | 17.8 ± 15.7 | 31.8 ± 18.8 | 37.9 ± 22.1 |
|  | Self-Efficacy [6] | 0.80 | 0.80 | 0.76 | 67.5 ± 16.0 | 66.1 ± 17.9 | 66.0 ± 16.9 |
| Offensive Behaviour | Sexual Harassment [1] | – | – | – | 2.9% | 0.6% | 33.6% |
|  | Threats of Violence [1] | – | – | – | 7.8% | 1.5% | 11.2% |
|  | Physical Violence [1] | – | – | – | 3.9% | 0.2% | 3.7% |
|  | Bullying [1] | – | – | – | 8.3% | 1.0% | 23.1% |
|  | Unpleasant Teasing [1] | – | – | – | 8.3% | 5.2% | 19.6% |
|  | Conflicts and Quarrels [1] | – | – | – | 51.2% | 5.8% | 70.5% |
|  | Gossip and Slander [1] | – | – | – | 38.9% | 5.3% | 37.9% |
| DK; Denmark version, PT; Portuguese version, GR; Greek version, SD; standard deviation.  a Prevalence proportions for the single items of the domain “offensive behaviour”.  Number in brackets [ ] refers to the number of items. | | | | | | | |

**Table 4.** Exploratory factor analysis of items in the Demands at Work Dimension (n=652) of COPSOQ II (long version): loadings for each factor and each item in the scale after varimax rotation and the factor extraction using principal components.

|  | Factors | | | | |
| --- | --- | --- | --- | --- | --- |
| **Scales - Items** | 1 | 2 | 3 | 4 | 5 |
| **Quantitative Demands** |  |  |  |  |  |
| [QD1] Is your workload unevenly distributed so it piles up? | 0.270 | 0.133 | **0.612** | 0.008 | -0.015 |
| [QD2] How often do you not have time to complete all your work tasks? | 0.075 | 0.061 | **0.849** | 0.191 | 0.067 |
| [QD3] Do you get behind with your work? | 0.079 | 0.099 | **0.724** | 0.278 | 0.029 |
| [QD4] Do you have enough time for your work tasks? | 0.052 | 0.051 | **-0.708** | 0.130 | 0.078 |
| **Work Pace** |  |  |  |  |  |
| [WP1] Do you have to work very fast? | **0.806** | -0.015 | -0.112 | -0.072 | 0.127 |
| [WP2] Do you work at a high pace throughout the day? | **0.742** | 0.074 | 0.217 | -0.025 | -0.121 |
| [WP3] Is it necessary to keep working at a high pace? | **0.794** | 0.058 | 0.278 | 0.160 | -0.010 |
| **Cognitive Demands** |  |  |  |  |  |
| [CD1] Do you have to keep your eyes on lots of things while you work? | **0.670** | 0.095 | 0.063 | **0.401** | 0.177 |
| [CD2] Does your work require that you remember a lot of things? | **0.488** | 0.122 | -0.099 | **0.487** | 0.310 |
| [CD3] Does your work demand that you are good at coming up with new ideas? | 0.038 | 0.158 | 0.112 | **0.819** | 0.046 |
| [CD4] Does your work require you to make difficult decisions? | 0.099 | 0.247 | 0.145 | **0.788** | -0.018 |
| **Emotional Demands** |  |  |  |  |  |
| [ED1] Does your work put you in emotionally disturbing situations? | -0.069 | **0.670** | 0.235 | 0.174 | 0.212 |
| [ED2] Do you have to relate to other people’s personal problems as part of your work? | 0.033 | **0.839** | -0.058 | 0.161 | 0.043 |
| [ED3] Is your work emotionally demanding? | 0.177 | **0.801** | -0.035 | 0.100 | 0.299 |
| [ED4] Do you get emotionally involved in your work? | 0.082 | **0.700** | 0.148 | 0.120 | 0.209 |
| **Demands for Hiding Emotions** |  |  |  |  |  |
| [HE1] Are you required to treat everyone equally, even if you do not feel like it? | 0.005 | 0.087 | 0.010 | 0.074 | **0.834** |
| [HE2] Does your work require that you hide your feelings? | 0.176 | 0.311 | -0.051 | 0.031 | **0.611** |
| [HE3] Are you required to be kind and open towards everyone – regardless of how they behave towards you? | -0.013 | 0.240 | 0.021 | 0.008 | **0.696** |
| Five factors explaining 64.0% of the total variance; Kaiser-Meyer-Olkin (KMO) = 0.797; Bartlett’s Test of Sphericity: *p* < 0.001. Bold values indicate factor loading of greater than 0.4. | | | | | |

**Table 5.** Exploratory factor analysis of items in the Work Organization and Job Contents (n=652) of COPSOQ II (long version): loadings for each factor and each item in the scale after varimax rotation and the factor extraction using principal components.

|  | Factors | | | | |
| --- | --- | --- | --- | --- | --- |
| **Scales - Items** | 1 | 2 | 3 | 4 | 5 |
| **Influence at Work** |  |  |  |  |  |
| [IN1] Do you have a large degree of influence concerning your work? | 0.122 | 0.235 | **0.706** | 0.044 | -0.165 |
| [IN2] Do you have a say in choosing who you work with? | 0.283 | -0.042 | **0.747** | -0.120 | -0.161 |
| [IN3] Can you influence the amount of work assigned to you? | 0.266 | 0.194 | **0.700** | 0.083 | 0.162 |
| [IN4] Do you have any influence on what you do at work? | 0.071 | 0.547 | **0.481** | 0.351 | 0.283 |
| **Possibilities for Development** |  |  |  |  |  |
| [PD1] Does your work require you to take the initiative? | -0.036 | **0.740** | 0.334 | 0.194 | 0.078 |
| [PD2] Do you have the possibility of learning new things through your work? | 0.343 | **0.695** | -0.018 | 0.020 | -0.155 |
| [PD3] Can you use your skills or expertise in your work? | 0.330 | **0.713** | 0.140 | 0.097 | -0.076 |
| [PD4] Does your work give you the opportunity to develop your skills? | 0.553 | **0.613** | 0.108 | 0.011 | -0.190 |
| **Variation of Work** |  |  |  |  |  |
| [VA1] Is your work varied? | **0.497** | **0.575** | 0.229 | -0.221 | -0.174 |
| [VA2] Do you have to do the same thing over and over again? | 0.013 | -0.121 | -0.100 | -0.192 | **-0.881** |
| **Meaning of Work** |  |  |  |  |  |
| [MW1] Is your work meaningful? | **0.540** | **0.471** | -0.100 | 0.362 | 0.171 |
| [MW2] Do you feel that the work you do is important? | **0.494** | 0.268 | -0.025 | 0.538 | -0.035 |
| [MW3] Do you feel motivated and involved in your work? | **0.747** | 0.303 | 0.186 | 0.062 | -0.051 |
| **Commitment to the Workplace** |  |  |  |  |  |
| [CW1] Do you enjoy telling others about your place of work? | **0.732** | 0.117 | **0.424** | -0.054 | -0.051 |
| [CW2] Do you feel that your place of work is of great importance to you? | **0.782** | 0.171 | 0.317 | 0.065 | 0.044 |
| [CW3] Would you recommend a good friend to apply for a position at your workplace? | **0.668** | 0.153 | 0.174 | 0.279 | 0.078 |
| [CW4] How often do you consider looking for work elsewhere? | -0.057 | -0.028 | -0.029 | **-0.842** | 0.196 |
| Five factors explaining 68.9% of the total variance; Kaiser-Meyer-Olkin (KMO) = 0.894; Bartlett’s Test of Sphericity: *p* < 0.001. Bold values indicate factor loading of greater than 0.4. | | | | | |

**Table 6.** Exploratory factor analysis of items in the Interpersonal Relations and Leadership (n=652) of COPSOQ II (long version): loadings for each factor and each item in the scale after varimax rotation and the factor extraction using principal components.

|  | Factors | | | | | |  |
| --- | --- | --- | --- | --- | --- | --- | --- |
| **Scales - Items** | 1 | 2 | 3 | 4 | 5 | 6 | |
| **Predictability** |  |  |  |  |  |  | |
| [PR1] At your place of work, are you informed well in advance concerning for example important decisions, changes, or plans for the future? | 0.283 | 0.218 | **0.534** | 0.221 | 0.054 | -0.088 | |
| [PR2] Do you receive all the information you need in order to do your work well? | **0.405** | 0.163 | 0.152 | 0.053 | **-0.443** | -0.128 | |
| **Recognition-Rewards** |  |  |  |  |  |  | |
| [RE1] Is your work recognized and appreciated by the management? | 0.306 | 0.157 | **0.840** | 0.079 | -0.117 | -0.107 | |
| [RE2] Does the management at your workplace respect you? | 0.352 | 0.094 | **0.819** | 0.102 | -0.065 | -0.178 | |
| [RE3] Are you treated fairly at your workplace? | 0.285 | 0.222 | **0.770** | 0.206 | -0.143 | -0.124 | |
| **Role Clarity** |  |  |  |  |  |  | |
| [CL1] Does your work have clear objectives? | 0.045 | 0.114 | 0.220 | **0.837** | -0.216 | 0.013 | |
| [CL2] Do you know exactly which areas are your responsibility? | -0.007 | 0.176 | 0.186 | **0.845** | -0.202 | -0.059 | |
| [CL3] Do you know exactly what is expected of you at work? | 0.175 | 0.181 | 0.028 | **0.637** | 0.074 | -0.326 | |
| **Role Conflicts** |  |  |  |  |  |  | |
| [CO1] Do you do things at work, which are accepted by some people but not by others? | 0.034 | -0.099 | -0.040 | -0.084 | **0.826** | -0.047 | |
| [CO2] Are contradictory demands placed on you at work? | -0.065 | 0.014 | -0.056 | -0.159 | **0.826** | 0.197 | |
| [CO3] Do you sometimes have to do things, which ought to have been done in a different way? | -0.116 | -0.031 | -0.116 | -0.007 | 0.277 | **0.814** | |
| [CO4] Do you sometimes have to do things, which seem to be unnecessary? | -0.036 | -0.026 | -0.216 | -0.200 | -0.044 | **0.811** | |
| **Quality of Leadership** |  |  |  |  |  |  | |
| [QL1] - makes sure that the individual member of staff has good development opportunities? | **0.844** | 0.002 | 0.103 | -0.115 | -0.073 | 0.074 | |
| [QL2] - gives high priority to job satisfaction? | **0.810** | 0.061 | 0.207 | -0.111 | -0.131 | 0.026 | |
| [QL3] - is good at work planning? | **0.613** | 0.235 | 0.212 | 0.342 | -0.023 | 0.002 | |
| [QL4] - is good at solving conflicts? | **0.664** | 0.252 | 0.266 | 0.296 | 0.125 | -0.033 | |
| **Social Support from Colleagues** |  |  |  |  |  |  | |
| [SC1] How often do you get help and support from your colleagues? | 0.157 | **0.742** | 0.009 | 0.264 | -0.018 | 0.055 | |
| [SC2] How often are your colleagues willing to listen to your problems at work? | 0.078 | **0.750** | 0.168 | 0.283 | -0.047 | 0.081 | |
| [SC3] How often do your colleagues talk with you about how well you carry out your work? | 0.244 | **0.636** | 0.087 | -0.128 | 0.157 | -0.057 | |
| **Social Support from Supervisors** |  |  |  |  |  |  | |
| [SS1] How often is your nearest superior willing to listen to your problems at work? | **0.704** | 0.201 | 0.165 | 0.069 | -0.021 | -0.207 | |
| [SS2] How often do you get help and support from your nearest superior? | **0.724** | 0.169 | 0.211 | 0.100 | -0.063 | -0.093 | |
| [SS3] How often does your nearest superior talk with you about how well you carry out your work? | **0.619** | 0.297 | 0.307 | 0.116 | 0.092 | -0.161 | |
| **Social Community at Work** |  |  |  |  |  |  | |
| [SW1] Is there a good atmosphere between you and your colleagues? | 0.116 | **0.732** | 0.182 | 0.134 | -0.159 | 0.018 | |
| [SW2] Is there good co-operation between the colleagues at work? | 0.061 | **0.823** | 0.170 | 0.155 | -0.155 | -0.063 | |
| [SW3] Do you feel part of a community at your place of work? | 0.225 | **0.733** | 0.074 | -0.037 | -0.038 | -0.196 | |
| Five factors explaining 68.3% of the total variance; Kaiser-Meyer-Olkin (KMO) = 0.862; Bartlett’s Test of Sphericity: *p* < 0.001. Bold values indicate factor loading of greater than 0.4. | | | | | | |  |

**Table 7.** Exploratory factor analysis of items in the Work-individual Interface (n=652) of COPSOQ II (long version): loadings for each factor and each item in the scale after varimax rotation and the factor extraction using principal components.

|  | Factors | | | |
| --- | --- | --- | --- | --- |
| **Scales - Items** | 1 | 2 | 3 | 4 |
| **Job Insecurity** |  |  |  |  |
| [JI1] Are you worried about becoming unemployed? | -0.035 | -0.052 | 0.358 | **0.687** |
| [JI2] Are you worried about new technology making you redundant? | -0.159 | -0.185 | 0.579 | **0.571** |
| [JI3] Are you worried about it being difficult for you to find another job if you became unemployed? | 0.066 | -0.130 | -0.146 | **0.749** |
| [JI4] Are you worried about being transferred to another job against your will? | 0.064 | -0.039 | -0.022 | **0.725** |
| **Job Satisfaction** |  |  |  |  |
| [JS1] - your work prospects? | 0.012 | **0.676** | -0.115 | -0.106 |
| [JS2] - the physical working conditions? | 0.093 | **0.616** | -0.438 | -0.025 |
| [JS3] - the way your abilities are used? | -0.049 | **0.800** | 0.057 | -0.131 |
| [JS4] -your job as a whole, everything taken into consideration? | -0.027 | **0.839** | 0.093 | -0.021 |
| **Work-Family Conflict** |  |  |  |  |
| [WF1] Do you often feel a conflict between your work and your private life, making you want to be in both places at the same time? | **0.726** | 0.044 | 0.053 | 0.039 |
| [WF2] Do you feel that your work drains so much of your energy that it has a negative effect on your private life? | **0.823** | -0.047 | -0.092 | 0.021 |
| [WF3] Do you feel that your work takes so much of your time that it has a negative effect on your private life? | **0.877** | -0.024 | 0.097 | 0.014 |
| [WF4] Do your friends or family tell you that you work too much? | **0.741** | 0.015 | 0.271 | -0.010 |
| **Family-Work Conflict** |  |  |  |  |
| [FW1] Do you feel that your private life takes so much of your energy that it has a negative effect on your work? | 0.201 | -0.020 | **0.842** | 0.005 |
| [FW2] Do you feel that your private life takes so much of your time that it has a negative effect on your work? | 0.155 | -0.015 | **0.852** | 0.018 |
| Five factors explaining 64.4% of the total variance; Kaiser-Meyer-Olkin (KMO) = 0.677; Bartlett’s Test of Sphericity: *p* < 0.001. Bold values indicate factor loading of greater than 0.4. | | | | |

**Table 8.** Exploratory factor analysis of items in the Values in the Workplace (n=652) of COPSOQ II (long version): loadings for each factor and each item in the scale after varimax rotation and the factor extraction using principal components.

|  | Factors | | | |
| --- | --- | --- | --- | --- |
| **Scales - Items** | 1 | 2 | 3 | 4 |
| **Mutual Trust between Employees** |  |  |  |  |
| [TE1] Do the employees withhold information from each other? | -0.174 | -0.191 | 0.036 | **-0.857** |
| [TE2] Do the employees withhold information from the management? | -0.167 | -0.212 | -0.053 | **-0.832** |
| [TE3] Do the employees in general trust each other? | 0.070 | **0.623** | -0.177 | -0.233 |
| **Trust Regarding Management** |  |  |  |  |
| [TM1] Does the management trust the employees to do their work well? | 0.295 | **0.795** | -0.025 | 0.086 |
| [TM2] Can you trust the information that comes from the management? | **0.703** | 0.283 | 0.091 | 0.015 |
| [TM3] Does the management withhold important information from the employees? | -0.133 | **-0.630** | -0.019 | 0.317 |
| [TM4] Are the employees able to express their views and feelings? | **0.629** | 0.316 | 0.080 | -0.253 |
| **Justice** |  |  |  |  |
| [JU1] Are conflicts resolved in a fair way? | **0.460** | **0.590** | 0.053 | -0.220 |
| [JU2] Are employees appreciated when they have done a good job? | **0.839** | 0.175 | 0.073 | -0.144 |
| [JU3] Are all suggestions from employees treated seriously by the management? | **0.837** | 0.138 | 0.086 | -0.254 |
| [JU4] Is the work distributed fairly? | **0.813** | 0.022 | 0.020 | -0.270 |
| **Social Inclusiveness** |  |  |  |  |
| [SI1] Are men and women treated equally at your workplace? | **0.576** | 0.123 | 0.213 | 0.147 |
| [SI2] Is there space for employees of a different race and religion? | 0.259 | -0.056 | **0.632** | 0.015 |
| [SI3] Is there space for elderly employees? | 0.068 | -0.164 | **0.838** | -0.097 |
| [SI4] Is there space for employees with various illnesses or disabilities? | 0.013 | 0.085 | **0.844** | 0.050 |
| Five factors explaining 64.6% of the total variance; Kaiser-Meyer-Olkin (KMO) = 0.823; Bartlett’s Test of Sphericity: *p* < 0.001. Bold values indicate factor loading of greater than 0.4. | | | | |

**Table 9.** Exploratory factor analysis of items in the Health and Well-being (n=652) of COPSOQ II (long version): loadings for each factor and each item in the scale after varimax rotation and the factor extraction using principal components.

|  | Factors | | | | | |
| --- | --- | --- | --- | --- | --- | --- |
| **Scales - Items** | 1 | 2 | 3 | 4 | 5 | 6 |
| **General Health Perception** |  |  |  |  |  |  |
| [GH1] In general, would you say your health is: | -0.204 | -0.130 | **0.464** | 0.147 | 0.111 | 0.309 |
| **Sleeping Problems** |  |  |  |  |  |  |
| [SL1] How often have you slept badly and restlessly | **0.477** | 0.128 | -0.042 | 0.071 | **0.466** | -0.389 |
| [SL2] How often have you found it hard to go to sleep? | 0.223 | 0.158 | 0.242 | 0.085 | 0.317 | **0.611** |
| [SL3] How often have you woken up too early and not been able to get back to sleep? | 0.069 | 0.154 | 0.279 | -0.019 | **0.786** | -0.117 |
| [SL4] How often have you woken up several times and found it difficult to get back to sleep? | 0.125 | 0.194 | 0.239 | -0.033 | **0.795** | -0.081 |
| **Burnout** |  |  |  |  |  |  |
| [BO1] How often have you felt worn out? | **0.827** | 0.062 | 0.081 | -0.054 | 0.130 | 0.164 |
| [BO2] How often have you been physically exhausted? | **0.817** | 0.181 | 0.161 | -0.127 | 0.075 | 0.158 |
| [BO3] How often have you been emotionally exhausted? | **0.625** | 0.362 | 0.282 | -0.094 | 0.092 | -0.139 |
| [BO4] How often have you felt tired? | **0.741** | 0.212 | 0.272 | -0.122 | -0.030 | 0.000 |
| **Stress** |  |  |  |  |  |  |
| [ST1] How often have you had problems relaxing? | **0.519** | 0.388 | **0.404** | -0.047 | 0.218 | -0.201 |
| [ST2] How often have you been irritable? | **0.450** | 0.351 | **0.481** | -0.023 | 0.116 | -0.202 |
| [ST3] How often have you been tense? | **0.589** | 0.288 | **0.442** | -0.038 | 0.130 | -0.112 |
| [ST4] How often have you been stressed? | **0.684** | 0.324 | 0.242 | -0.079 | 0.196 | 0.017 |
| **Depressive Symptoms** |  |  |  |  |  |  |
| [DS1] How often have you felt sad? | 0.194 | 0.271 | **0.603** | 0.024 | 0.165 | -0.279 |
| [DS2] How often have you lacked self-confidence? | 0.145 | 0.104 | **0.622** | -0.249 | 0.273 | -0.120 |
| [DS3] How often have you had a bad conscience or felt guilty? | 0.192 | 0.224 | **0.561** | -0.082 | 0.225 | -0.158 |
| [DS4] How often have you lacked interest in everyday things? | 0.260 | 0.105 | **0.698** | -0.033 | 0.167 | 0.248 |
| **Somatic Stress** |  |  |  |  |  |  |
| [SO1] How often have you had stomachache? | 0.370 | 0.340 | 0.100 | -0.207 | **0.451** | 0.254 |
| [SO2] How often have you had a headache? | 0.236 | **0.585** | 0.074 | -0.262 | 0.387 | -0.009 |
| [SO3] How often have you had palpitations? | 0.243 | **0.547** | 0.025 | -0.194 | 0.361 | -0.133 |
| [SO4] How often have you had tension in various muscles? | **0.478** | **0.505** | 0.117 | -0.108 | 0.217 | -0.177 |
| **Cognitive Stress** |  |  |  |  |  |  |
| [CS1] How often have you had problems concentrating? | 0.284 | **0.758** | 0.078 | -0.133 | 0.115 | 0.042 |
| [CS2] How often have you found it difficult to think clearly? | 0.214 | **0.773** | 0.196 | -0.088 | 0.107 | -0.176 |
| [CS3] How often have you had difficulty in taking decisions? | 0.082 | **0.752** | 0.279 | -0.161 | 0.089 | -0.042 |
| [CS4] How often have you had difficulty with remembering? | 0.206 | **0.692** | 0.276 | -0.147 | 0.010 | 0.134 |
| **Self-Efficacy** |  |  |  |  |  |  |
| [SE1] I am always able to solve difficult problems, if I try hard enough. | 0.258 | -0.009 | -0.119 | 0.173 | -0.043 | **0.824** |
| [SE2] If people work against me, I find a way of achieving what I want. | 0.053 | 0.032 | -0.092 | **0.401** | -0.020 | **0.665** |
| [SE3] It is easy for me to stick to my plans and reach my objectives. | -0.089 | -0.084 | -0.040 | **0.740** | -0.027 | -0.025 |
| [SE4] I feel confident that I can handle unexpected events. | -0.125 | -0.176 | -0.014 | **0.825** | -0.082 | 0.050 |
| [SE5] When I have a problem, I can usually find several ways of solving it. | -0.082 | -0.192 | -0.077 | **0.791** | 0.000 | 0.121 |
| [SE6] Regardless of what happens, I usually manage. | -0.019 | -0.123 | -0.156 | **0.714** | -0.023 | 0.193 |
| Five factors explaining 64.3% of the total variance; Kaiser-Meyer-Olkin (KMO) = 0.911; Bartlett’s Test of Sphericity: p < 0.001. Bold values indicate factor loading of greater than 0.4. | | | | | | |

**Table 10.** Exploratory factor analysis of items in the Offensive Behaviour (n=652) of COPSOQ II (long version): loadings for each factor and each item in the scale after varimax rotation and the factor extraction using principal components.

|  | Factors | |
| --- | --- | --- |
| **Scales - Items** | 1 | 2 |
| **Sexual Harassment** |  |  |
| [SH] Have you been exposed to undesired sexual attention at your workplace during the last 12 months? | **0.900** | 0.209 |
| **Threats of Violence** |  |  |
| [TV] Have you been exposed to threats of violence at your workplace during the last 12 months? | 0.130 | **0.793** |
| **Physical Violence** |  |  |
| [PV] Have you been exposed to physical violence at your workplace during the last 12 months? | 0.007 | **0.738** |
| **Bullying** |  |  |
| [BU] Have you been exposed to bullying at your workplace during the last 12 months? | **0.917** | 0.247 |
| **Unpleasant Teasing** |  |  |
| [UT] Have you been exposed to unpleasant teasing at your workplace during the last 12 months? | **0.903** | 0.237 |
| **Conflicts and Quarrels** |  |  |
| [CQ] Have you been involved in quarrels or conflicts at your workplace during the last 12 months? | 0.302 | **0.421** |
| **Gossip and Slander** |  |  |
| [GS] Have you been exposed to gossip and slander at your workplace during the last 12 months? | **0.914** | -0.061 |
| Five factors explaining 70.4% of the total variance; Kaiser-Meyer-Olkin (KMO) = 0.816; Bartlett’s Test of Sphericity: *p* < 0.001. Bold values indicate factor loading of greater than 0.4. | | |
